# The potential of mobile health clinics in chronic disease prevention and health promotion in universal healthcare systems. An on-field experiment

**DOI:** 10.1186/s12939-020-01174-8

**Published:** 2020-05-01

**Authors:** Chiara Bertoncello, Silvia Cocchio, Marco Fonzo, Silvia Eugenia Bennici, Francesca Russo, Giovanni Putoto

**Affiliations:** 1 Hygiene and Public Health Unit, DCTVSP Department of Cardiac Thoracic and Vascular Sciences and Public Health, University of Padua, Via Loredan 18, 35131 Padova (PD), Italy; 2 Organizational Unit Prevention and Public Health, Venice, Veneto Region Italy; 3 Doctors with Africa CUAMM, Padua, Italy

**Keywords:** Mobile health units, Noncommunicable diseases, Health promotion, Primary health care, Healthcare inequalities

## Abstract

**Background:**

Mobile health clinics (MHCs) are recognized to facilitate access to healthcare services, especially in disadvantaged populations. Notwithstanding that in Europe a wide-ranging background in mobile screening units for cancer is shared, evidences about MHCs targeting also at other non-communicable diseases (NCDs) in universal health coverage systems are scarce. The aim of this study was to describe the population attracted with a MHC initiative and to assess the potential of this tool in prevention and control of NCDs.

**Methods:**

Our MHC was set up in a railway wagon. Standard body measurements, finger-stick glucose, total cholesterol and blood pressure were recorded. Participants were asked about smoking, physical activity, diet, compliance to national cancer screening programmes and ongoing pharmacological treatment. One-to-one counselling was then provided.

**Results:**

Participants (*n* = 839) showed a higher prevalence of overweight/obesity, insufficient intake of vegetables, sedentary lifestyle, and a lower compliance to cancer screening compared with reference population. Our initiative attracted groups at higher risk, such as foreigners, men and people aged from 50 to 69. The proportion of newly diagnosed or uncontrolled disease exceeded 40% of participants for both hypertension and hypercholesterolemia (7% for diabetes). Adherence rate to counselling was 99.4%.

**Conclusions:**

The MHC was effective in attracting hard-to-reach groups and individuals who may have otherwise gone undiagnosed. MHCs can play a complementary role also in universal coverage health systems, raising self-awareness of unreached population and making access to primary health care easier.

## Key-points

- Mobile health clinics (MHCs) facilitate access to healthcare service.
- Scarce literature on MHCs for NCDs other than cancer in universal coverage systems.
- Our MHC detected undiagnosed conditions, bad lifestyles, lower compliance cancer screening.
- Effective in attracting foreigners, men, aged 50–69.
- MHCs can play a complementary role also in universal coverage systems.

## Introduction

Noncommunicable diseases (NCDs) – including cardiovascular diseases, cancer, chronic respiratory diseases and diabetes – are by far the leading cause of death according to the latest estimates. In 2016, they were responsible for 71% of all deaths globally. In Italy, NCDs account for 91% of all deaths, [1]. The prevalence of NCDs is expected to rise over the next decades due to the ageing of population and an increase of risk factors [2]. Lifestyle counselling activities, screening initiatives, management of risk factors and treatment of disabilities are intended to become even more predominant in planning public health strategies [3].

The burden of NCDs does not affect population in an equal manner. As a whole, European National Healthcare Systems (NHSs) seem to be effective in narrowing the gap due to health inequalities originating from socioeconomic status (SES), nationality and gender [4, 5] in terms of mortality rates [6]. However, European NHSs seem not to be equally effective in reducing risk factors: prevalence of smoking, overweight/obesity, unhealthy diet and physical inactivity is higher in most disadvantaged sections of the population [7].

Socioeconomic inequalities heavily affect the participation in screening campaigns and contribute to worse outcomes [8] Thus, any effort made to extend the benefits of screening to individuals who may have otherwise gone undiagnosed – or diagnosed at a late-stage – appear reasonable.

In this respect, mobile health clinics (MHCs) could make a significant contribution, facilitating the access to healthcare services by reducing issues related with transportation and avoiding long waiting times and complicated administrative procedures [9, 10]. MHCs are used in a wide range of low and middle-income countries [11] and in the United States (US), where they are monitored by the national programme *Mobile Health Map* [9]. In the US, they are shown to facilitate access for minority groups, to attract people who usually exhibit poorer healthcare-seeking behaviours such as male patients [11–13] and to improve patient adherence to therapy [9].

The idea of screening for NCDs with MHCs dates back to 1960 [14, 15] and the employment of mobile units for cancer screening is a consolidated practice in Europe and Italy [16]. Nonetheless, literature about the efficacy of MHCs specifically addressed to prevent and control NCDs – except for early detection of cancer – is lacking.

Mobile screening units are very effective in increasing community access to cancer screening [2]. In Italy, the so-called *‘mammography vans’* are commonly used within the national breast cancer screening program, both in association with fixed clinics and in exclusive use, particularly in regions with extended rural areas [17]. In addition to cancer screening, MHCs are often used in setting up information campaigns on the prevention and control of NCDs [18] or as research units with the aim of describing the prevalence of NCDs within the community and the level of chronicity management in the different parts of the country [19, 20]. The main difference between the mobile units for cancer screening and the MHC for NCDs lies not so much in in the way the services are delivered – in both cases through a mobile clinic – but rather in the strategy and purpose of use: while the former are used as a way of offering health services that are routinely implemented in a national screening program, services offered in MHCs for NCDs are to be considered as part of information and awareness-raising strategies. Services for the prevention and control of NCDs are provided in primary care services (such as general practitioners’ surgeries and prevention services), but a nationally shared framework is missing, and the service is provided on a case-by-case basis.

Although primary health care services are ‘offered to all’ (free or co-payment) in our context, ‘accessibility for all’ in real life may still pose a challenge due to issues – such as disparities in the socio-economic status – that are not directly addressed by the NHS. The access to quality primary care for all is a major concern also in Europe. Insufficient access to primary care is a defeat for the individual and the society, as it ultimately leads to an increase in the disease burden at both levels. There is a need for new strategies that can overcome barriers and provide effective, accessible and affordable primary care for all [21].

MHC could be a useful strategy to reach this goal, but more evidences are needed. When testing the validity and the efficacy of MHC in preventing and controlling NCDs, it is important to consider the national health system context. In this work, we illustrate an initiative that could be studied and considered as a best practice. A MHC initiative with the main purpose of providing screening for NCDs and counselling for health promotion and prevention was set up in Veneto Region, Italy in 2017. The aim of the present study was to (i) describe the population attracted with a MHC initiative in a Universal Health Coverage System; (ii) assess the potential contribution of this initiative in the prevention and the control of NCDs.

## Methods

The MHC initiative was funded and organized by the Regional Health Authority and a local non-governmental organization between November and December 2017. The MHC was set up in a dedicated railway wagon and visited the main train stations in the Veneto Region (Italy), whose population is about 5 million inhabitants. Access to the MHC was completely free of charge for attendants; the MHC was open from 9 AM to 7 PM (Mon-Sat) and from 9 AM to 2 PM on Sundays for a total of 21 days of service. Local media were used to raise awareness about the initiative.

Biometric screening and counselling were provided into two adjacent wagons. Standard body measurements including height, weight and waist circumference were recorded. Finger-stick glucose, total cholesterol and blood pressure were recorded. Participants were asked about gender, age, nationality, education, employment, smoking habit, physical activity, diet and compliance to the national cancer screening programmes against cervical, breast and colorectal cancer. Individuals were also asked about the use of medications for high blood pressure, diabetes or high cholesterol. Based on medical findings and patient’s medical history, participants were provided with counselling about smoking, physical activity, healthy diet and cancer screenings; when needed, they were referred to their general practitioner for further investigations. The staff consisted of medical doctors and nurses employed in the Italian National Health Service (or retired from it) and medical students. They agreed to participate on a voluntary basis and they did not receive any compensation for their time. All staff members had received a specific training on counselling prior to the start of the MHC initiative.

Sociodemographic, behavioural and health-related characteristics of participants were compared with the general population in the same Region. The following cardiovascular risk factors were considered: high blood pressure (≥ 140/90 mmHg); high blood total cholesterol (≥ 200 mg/dL); high fasting blood glucose (≥ 126 mg/dL); overweight and obesity (BMI ≥ 25 kg/m^2^); smoking. According with the self-reported physical activity and latest WHO *Global Recommendations on Physical Activity* [22], participants were classified as ‘*active’* if they reported at least 150 min of moderate-intensity activity or 75 min of vigorous activity per week; ‘*sedentary’* when they reported no physical activity; ‘*partially active’* if in the between. The number of fruits and vegetables portions consumed per day was classified in no servings; one or two servings; three or more servings.

The potential of this initiative was assessed in terms of: (i) proportion of newly diagnosed or uncontrolled disease; and (ii) rate of adherence to counselling service following the biometric screening procedures.

Quantitative analyses were conducted with IBM SPSS® Statistics v23; χ^2^ tests were performed to compare the study population with the reference population in terms of sociodemographic and health-related characteristics. *P* values were reported.

## Results

Individuals who participated in the initiative were 839. The median age was 55 years (Q_1_:38; Q_3_: 65). Sociodemographic, behavioural and health-related characteristics are shown in Table 1: 54.1% of attendants were males; foreigners were 16.6% while they are the 9.9% of the general population in the region. The proportion of overweight and sedentary lifestyle was higher than the general population, while the daily fruit and vegetable intake was lower, as well as the compliance to routine cancer screenings. On the other hand, the proportion of smokers was lower. The percentage of individuals who had received already a pharmacological treatment for hypertension, diabetes or hypercholesterolemia was lower compared with the general population. The study population was characterized by a more sedentary lifestyle compared with the reference population (23.0% vs. 15.2%). While females were more sedentary than males (26.3% vs. 16.9%, *p* < 0.01), men were more overweight (65.0% vs. 44.1%, *p* < 0.01) and used to eat less fruit and vegetables (74.1% vs. 65.9%, *p* = 0.03), as shown in Table 2. Among foreigners, the proportion of smokers and diet poor in vegetables was significantly higher than Italians (21.2% vs. 13.0%, *p* = 0.01 and 83.3% vs. 67.9%, *p* < 0.01, respectively). The obesity rate was the highest among people with secondary education (63.9%), while it was the lowest among people holding a university degree (46.4%). The prevalence of obesity was the highest among retired people, even though the prevalence of sedentary lifestyle was the lowest (13.8%). The highest proportion of smokers was recorded among unemployed (28.8%).

**Table 1:** Characteristics of participants of MHC initiative compared with general population living in the same region

		Study population		Reference Population		
Sociodemographic characteristics^a^		**n**	**%**	**n**	**%**	**χ2 test (p)**
**Gender**	Female	385	45,9%	2.512.962	51,2%	< 0.01
	Male	454	54,1%	2.394.567	48,8%	
**Age**	0–9 y	0	0,0%	435.947	8,88%	< 0.01
	10–19 y	22	2,6%	466.277	9,50%	
	20–29 y	117	14,0%	472.853	9,64%	
	30–39 y	78	9,4%	574.591	11,71%	
	40–49 y	101	12,1%	802.881	16,36%	
	50–59 y	195	23,4%	758.442	15,45%	
	60–69 y	192	23,0%	589.202	12,01%	
	70–79 y	96	11,5%	478.430	9,75%	
	80+ y	33	4,0%	328.906	6,70%	
**Nationality**	Italians	687	83,4%	4.422.052	90,1%	< 0.01
	Foreigners	137	16,6%	485.477	9,9%	
**Education**^**b**^	None or Primary School	75	9,4%	1.326.872	29,0%	< 0.01
	Lower Secondary School	159	19,9%	1.378.977	30,1%	
	High School	393	49,3%	1.450.833	31,7%	
	Degree	170	21,3%	421.682	9,2%	
**Employment**	Employed	394	50,3%	2.081.000	49,6%	< 0.01
	Unemployed	52	6,6%	151.000	3,6%	
	Inactive	337	43,0%	1.967.000	46,8%	
	Retired	221	28,2%	NA	NA	
	Student	97	12,4%	NA	NA	
	Housekeeper	19	2,4%	NA	NA	
Health-related conditions^c^		**n**	**%**	**n**	**%**	**χ2 test (p)**
**Smoking habit**	Smoker	114	16,4%	889	21,8%	< 0.01
	Ex-smoker	124	17,8%	841	20,6%	
	Non-smoker	457	65,8%	2.345	57,6%	
**Hypertension drug treatment**		104	14,9%	759	20,1%	< 0.01
**Diabetes drug treatment**		20	2,9%	146	3,6%	0.34
**Hypercholesterolemia drug treatment**		35	5,0%	792	24,8%	< 0.01
**BMI**	Underweight/Normal (< 25 kg/m2)	314	45,8%	2.402	59,2%	< 0.01
	Overweight (25–30 kg/m2)	242	35,3%	1.260	31,0%	
	Obese (> 30 kg/m2)	130	19,0%	393	9,8%	
**Physical activity**	Sedentary	135	23,0%	618	15,2%	< 0.01
	Partially active	99	16,8%	1.217	29,9%	
	Active	354	60,2%	2.231	54,9%	
**Daily Fruit and Vegetable Intake**	None	56	11,8%	70	1,7%	< 0.01
	1–2 servings	278	58,8%	1.794	44,0%	
	3+ servings	139	29,4%	2.211	54,3%	
**Compliance to national Breast Cancer Screening**^**d**^	Yes	152	80,9%	1.145	86,1%	0.15
**Compliance to national Cervical Cancer Screening**^**e**^	Yes	173	70,0%	1.453	90,9%	< 0.01
**Compliance to national Colorectal Cancer Screening**^**f**^	Yes	251	64,9%	1.277	77,2%	< 0.01

^a^ISTAT, Italian National Institute of Statistics; data warehouse updated on 1 January 2017

^b^ISTAT, Italian National Institute of Statistics; national census, 2011

^c^PASSI, national surveillance program, Veneto Region data warehouse, 2014–2017; only 18–69 years old included

^d^only target population included (females 50–69 y)

^e^only target population included (females 25–64 y)

^f^only target population included (both sexes 50–69 y)

**Table 2:** Distribution of modifiable risk factors by baseline characteristics

		Risk factors							
		Smokers		Overweight/obesity (BMI > 25)		Sedentary lifestyle		Less than 3 servings fruit/veg	
		**%**	**χ2 test (p)**	**%**	**χ2 test (p)**	**%**	**χ2 test (p)**	**%**	**χ2 test (p)**
Gender	Female	13.1%	0.18	44.1%	**< 0.01**	26.3%	**< 0.01**	65.9%	**0.03**
	Male	16.4%		65.0%		16.9%		74.1%	
Age	10–19 y	31.8%	**< 0.01**	14.3%	**< 0.01**	50.0%	**< 0.01**	75.0%	0.05
	20–29 y	15.4%		33.9%		23.5%		71.0%	
	30–39 y	23.1%		42.1%		19.0%		85.7%	
	40–49 y	15.0%		65.0%		27.9%		78.9%	
	50–59 y	19.7%		59.5%		25.3%		68.2%	
	60–69 y	10.5%		64.7%		18.0%		62.6%	
	70–79 y	6.3%		62.1%		9.5%		67.1%	
	80+ y	3.0%		60.6%		15.4%		79.2%	
Nationality	Italians	13.0%	**0.01**	55.5%	0.97	20.0%	0.14	67.9%	**< 0.01**
	Foreigners	21.2%		55.6%		26.5%		83.3%	
Education	None or Primary School	16.2%	0.31	58.1%	**0.02**	14.3%	0.57	70.6%	0.99
	Lower Seconday School	15.7%		63.9%		20.4%		70.1%	
	High School	15.9%		54.5%		22.5%		69.9%	
	Degree	10.1%		46.4%		21.1%		71.4%	
Employment	Employed	16.1%	**< 0.01**	58.0%	**< 0.01**	25.1%	**0.02**	71.8%	0.35
	Unemployed	28.8%		48.1%		21.1%		80.0%	
	Retired	7.3%		63.2%		13.8%		64.7%	
	Student	18.6%		34.0%		28.6%		71.4%	
	Houskeeper	5.3%		52.6%		22.2%		71.4%	

In Table 3, the proportion of newly detected cases and uncontrolled cases of the chronic conditions investigated is shown. A ‘*new case’* is defined by an out of range value following biometric screening without a pharmacological treatment, while ‘*uncontrolled cases’* are characterized by a previous start of pharmacological therapy. Patients with a new diagnosis of high blood pressure were 27.8% of the study population, while new cases of diabetes and hypercholesterolemia were 5.0 and 37.5%, respectively. Cases of uncontrolled hypertension reached 12.8% of the sample, while uncontrolled diabetes and hypercholesterolemia accounted for 2.0 and 2.7%, respectively.

**Table 3:**
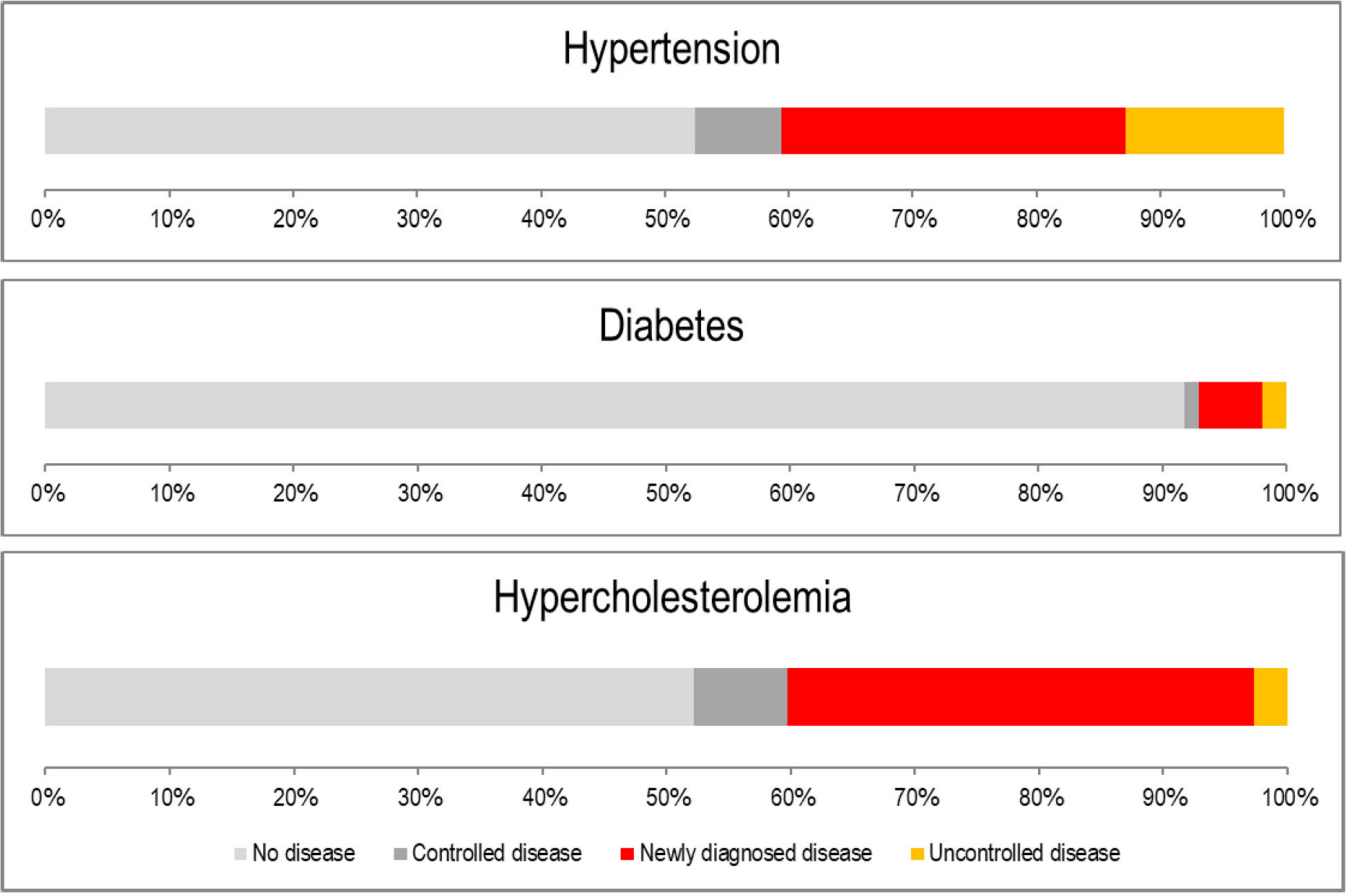
Distribution of newly diagnosed and uncontrolled conditions investigated

Out of 497 participants who were suggested to undergo at least one of the counselling services provided in the adjacent wagon, 494 agreed to receive the counselling (rate of adherence: 99.4%); 238 participants received counselling about smoking, 273 on cancer screenings, 352 about healthy diet and 294 about physical activity.

Overall, 319 participants (38%) were invited to contact their general practitioner; 6 (0,7%) were referred to the nearest emergency department because of a hypertensive or hyperglycaemic ongoing crisis.

## Discussion

Participants in our initiative showed substantial differences in sociodemographic, behavioural and health-related characteristics compared with the general population. The MHC initiative was able to address to individuals who showed more risk factors compared with the general population, consistently with other studies [9, 11, 12].

The proportion of males, 50–69-year-old individuals, foreigners and people with higher education was significantly higher than the general population.

The ability of attracting the male population (54.1% of participants) is consistent with other experiences [12, 13]. Usually men show lower access to traditional health care facilities than women [13]. This may be due to peculiar features of the MHC: waiting times are limited, opening hours extended and people are not expected to book an appointment or to request a leave from work.

Foreigners may experience further barriers: navigating the healthcare system could be a complex task to achieve; primary health services might be ignored although offered free of charge; linguistic, cultural or psychological barriers and intimidation by healthcare settings may limit the access [11]. As a result, foreigners tend to overuse emergency departments where access appear easier and more immediate [23, 24]. This situation unavoidably leads to an increase in healthcare costs for the inappropriate use of emergency departments and to a widening of health inequalities due to a lack of prevention and management of chronic diseases, that are estimated to affect four foreigners out of ten in Italy [25, 26]. According to our results, foreigners show more behavioural risk factors for NCDs: smoking, sedentary lifestyle and lower intake of fruit and vegetables. Many studies confirm that immigrants participate less than natives in organised cancer screenings [22–24] and eat less portions of fruits and vegetables [27, 28]. The prevalence of NCDs and their risk factors should not be underestimated in foreigners as our data and other studies suggest [29, 30]. In the long-term this scenario may even worsen in relation to the process of getting acquainted to western unhealthy dietary and voluptuary habits [31–33].

Overall, participants showed a higher prevalence of unhealthy behaviours such as an insufficient daily intake of fruit and vegetable, sedentary lifestyle, overweight and a lower compliance to cancer screening (except for screening against breast cancer) compared with the general population. However, the proportion of participants who had started already a pharmacological treatment for hypertension, diabetes or hypercholesterolemia was lower than the general population. This finding may be interpreted as a warning light of insufficient access to primary care facilities – where these conditions are usually primarily diagnosed – rather than an evidence of a better health status, especially in the light of a higher rate of risk factors.

As a direct consequence, our MHC initiative was able to diagnose a remarkable number of new cases or cases of uncontrolled disease. The prevalence of previously undetected hypertension (27.8%), hypercholesterolemia (37.5%) and diabetes (5.0%) was analogue to other comparable international experiences [34, 35]. This offered to participants the chance to get acquainted with their own health condition and encouraged them to seek primary health care for an appropriate long-term follow up [36–38]. The counselling activity about the importance of getting below the recommended targets could play a key role in increasing patients’ adherence to the therapy [9, 39].

MHCs were shown to be effective in improving health outcomes in the population, whether they are considered as ‘alternatives’ to more traditional healthcare models or not [21]. MHCs can reach cross-sections of the population that are at higher risk or stigmatized and help in identifying additional cases of NCDs: without these services, diagnoses and treatment would be delayed and subsequent management further complicated in more vulnerable groups [40, 41]. Findings from our MHC initiative definitely move in this direction: the proportion of newly diagnosed or uncontrolled disease, collectively, exceeded 40% of participants for both hypertension and hypercholesterolemia, although this percentage was significantly lower (7.1%) for diabetes. These results show how the MHCs bear an unexpressed potential.

Adherence to therapy and lifestyle changes have a pivotal role in the management of NCDs. Evidences show that MHCs are effective in sustaining patients to achieve these goals [21]: for example, screening and counselling services provided in the MHC described by Song et al. showed to be effective in lowering blood pressure in hypertensive patients [42]. Unfortunately, our MHC initiative was a *‘première’* in our context and data on follow-up visits were not available; however, the considerable proportion of new diagnoses and uncontrolled disease in our study together suggest a high value of this tool and brief counselling activities are shown to be effective also when provided only on a single occasion [43].

In addition, results are overwhelmingly encouraging in the light of the fact that the rate of adherence to counselling interventions proposed was very close to 100% (99.4%).

In 38% of cases, participants were suggested to consult their general practitioner for a comprehensive and long-term management of chronicity. Unfortunately, we could not verify if participants actually consulted their general practitioner after the counselling service – data-linkage was not possible – but past studies showed the ability of MHCs in connecting community members with both medical and social services and the efficacy in reducing emergency department and hospital admissions for NCDs and their complications [21, 44].

In our study, adherence to counselling was very high. This may imply a positive impact given the fact that: (i) one-to-one counselling activities are strongly recommended to increase adherence to cancer screening [45]; (ii) counselling was shown to improve dietary and physical activity behaviors and reduce smoking habit, cholesterol levels, blood pressure, weight, glucose levels, and incidence of diabetes [46, 47].

The key of the success for a high adherence rate to counselling may be due to several factors: (i) by providing a more intimate, welcoming and less intimidating environment, the MHC put patients at the heart of the process, bringing healthcare into community spaces familiar to patients, allowing them to feel the sense of a more complete involvement and self-efficacy [11, 43]; (ii) all services provided were completely free; (iii) opening hours were broader than primary care ‘traditional’ facilities; (iv) the counselling was offered on the same occasion of biometric screening – the two services were provided in two adjacent wagons. Where practicable, it makes sense to integrate the provision of multiple services to enhance participation [48].

There is a strong evidence that reducing structural barriers and facilitating access to health care services – by reducing the distance between the service delivery settings and the target population, or changing service hours to meet patients’ needs – are successful strategies that increase the adherence rate to screening for breast, cervical and colorectal cancer [45]. For this reason, MHCs for early detection of cancer are commonly used in Europe. In Italy, mobile vans for mammography have been used extensively in residential communities. Worldwide, cancer screening has been the most common service provided by MHC, but they are not the only ones: the services offered by MHCs are manifold, from primary to tertiary care [21]. The preventive services include screening for HIV and sexually transmitted diseases, ophthalmological diseases, cardiovascular conditions and diabetes, but also health promotion activities such as vaccinations or counselling or initiating preventative care, managing chronic diseases and enabling self-efficacy [9, 21].

Given the success of mobile units for cancer screening, it makes sense to extend the use of MHCs for other NCDs, implementing screening and counselling activities specifically addressed to prevention and management of chronicity. Khanna and colleagues showed how generally people do not consider MHC as substitute for primary healthcare facilities [21]; in this regard, our experience suggests that MHCs in our context can complement primary care by intercepting unexpressed needs. To achieve this goal, MHCs should be extended to reach even more remote rural areas and not only cities, resorting also to means of transportation other than train.

MHCs offer more opportunities for underserved populations to assess their health conditions and learn how to manage their health properly, by facilitating access to healthcare [42]. MHCs represent an extraordinary resource for those who would not otherwise ask for assistance to a health centre, delaying both diagnosis and treatment. The core of the management of chronic conditions is to support adherence to necessary medication and lifestyle changes: evidences suggest that MHCs are effective in helping patients meet these challenges [9].

Our MHC initiative occurred in the main train stations of the region. This may imply that individuals who do not live in urban areas or do not use trains were under-represented in the sample. Data were not available for all participants in every single required fields, leaving a slightly different denominator for each computation due to missing values. Some data were self-reported by the participants and we had no tools to validate them. Because of the *white coat effect,* having relied on a single measure of blood pressure may have led to an overestimation of the prevalence of hypertension among those screened. Unlike many other documented experiences, our MHC initiative was a one-time event, making follow up and monitoring of outcomes not possible. However, brief counselling activities are shown to be efficient and cost-effective in improving health status also when provided only on a single occasion [43].

## Conclusions

Although MHCs could be considered redundant in a universal health coverage system as there is in Italy, our findings challenge this concept. Also in settings where primary care services are free of access and free of charge, MHCs can have a complementary role making a substantial contribution in reducing sociodemographic inequalities [9]. MHCs can intercept those cross-sections of the population which are usually difficult to reach, providing more easily accessible care and serving as a help in navigating traditional healthcare facilities. Currently in Italy, a national-based screening programme for NCDs other than cancer has not been implemented. The evaluation and the management of risk factors are carried out by general practitioners, each one individually. Our findings suggest that MHCs could be considered as a powerful and complementary tool in providing screening and counselling for NCDs (acceptance rate of receiving counselling was 99.4%) and further extending the proportion of people that can be reached.

Despite there being relatively few studies, the literature is able to provide a solid degree of evidence necessary for quantitative and qualitative assessments of the role of MHCs in reducing the impact of NCDs, not only through cancer screening.

Since the main difference between the MHC for cancer screening and the MHC for CDs lies not so much in in the way the services are delivered – in both cases through a mobile clinic – but rather in the strategy and purpose of use, an important impact in reducing morbidity and mortality of other NCDs can be expected through the adoption of this service delivery strategy, following the success of MHC-based strategies for cancer screening. This work is intended to be a valuable support in building evidence in this regard.

## Data Availability

The datasets used and/or analysed during the current study are available from the corresponding author on reasonable request.
